# Factitious Hyperthyroidism: A Case of Levothyroxine Misuse Induced by Occupational Stress

**DOI:** 10.1192/j.eurpsy.2026.11852

**Published:** 2026-07-31

**Authors:** N. Gannoun, F. Ben Abdesslem, G. Saad, I. Halloul, N. Belhadj, F. Chelly, C. Sridi, A. Fki, M. Maoua, Y. Hasni

**Affiliations:** 1 Occupational Medicine Department, Sahloul University Hospital; 2 Faculty of Medicine of Sousse, University of Sousse; 3 Sousse, Research laboratory (LR19SP03); 4 Department of Endocrinology, University Hospital of Farhat Hached Sousse, Sousse, Tunisia

## Abstract

**Introduction:**

Hyperthyroidism is an endocrine disorder Resulting from excessive thyroid hormone levels. While autoimmune thyroid disease and nodular pathology are the most common causes, factitious hyperthyroidism induced by exogenous thyroid hormone intake represents a diagnostic challenge.

**Objectives:**

To report a case of factitious hyperthyroidism due to levothyroxine misuse, highlighting its professional and psychiatric implications.

**Methods:**

A descriptive case study integrating clinical, biochemical, and imaging data with a comprehensive psychosocial assessment.

**Results:**

A 38-year-old woman with no relevant personal medical history, was admitted for severe biochemical hyperthyroidism (FT4: 80 pmol/L; TSH <0.001 mUI/L). She presented with low-grade fever and an 8-kg unintentional weight loss over three months, without abnormal physical findings. TSH receptor antibodies were negative, inflammatory markers were normal (CRP: 6 mg/L; ESR: 4 mm), and cervical ultrasound suggested acute thyroiditis with concordant negative scintigraphy. The inconsistency between clinical, biochemical, and imaging findings prompted a psychosocial evaluation, during which the patient disclosed deliberate daily intake of levothyroxine for three months. This behavior was precipitated by persistent occupational stress and interpersonal conflicts at work, and was triggered by the refusal of her annual leave request, perceived as intolerable.

**Conclusions:**

This case illustrates how occupational stressors can precipitate maladaptive coping behaviors, manifesting as factitious endocrine disorders Factitious levothyroxine intake represented the patient’s only means of obtaining sick leave. This case highlights the necessity of providing listening and psychological support within the workplace to identify vulnerable individuals at risk of maladaptive coping.

**Disclosure of Interest:**

None Declared

